# Supplementary material for: Modeling large fluctuations of thousands of clones during hematopoiesis: The role of stem cell self-renewal and bursty progenitor dynamics in rhesus macaque
Source: PLoS Comput Biol. 2018 Oct 18;14(10):e1006489. doi: 10.1371/journal.pcbi.1006489 (PMC6218102; doi:10.1371/journal.pcbi.1006489)
Supplement: S1 Appendix — (PDF) [file pcbi.1006489.s001.pdf]

S1 Appendix: Mathematical Appendices for "Modeling large fluctuations of thousands of clones during hematopoiesis: the role of stem cell self-renewal and bursty progenitor dynamics in rhesus macaque"

Song Xu[1], Sanggu Kim[2], Irvin S. Y. Chen[3], Tom Chou[4,*]

1 Center for Biomedical Informatics Research, Dept. of Medicine, Stanford University, Stanford, CA 94305, USA

2 Dept. of Veterinary Biosciences, Ohio State Univ., Columbus, OH 41320, USA

3 UCLA AIDS Institute and Dept. of Microbiology, Immunology, and Molecular Genetics, UCLA, Los Angeles, CA 90095, USA

4 Dept. of Mathematics, UCLA, Los Angeles, CA 90095-1555, USA

A Stochastic evolution of HSC clone sizes

To solve Eq. (6) in the main text for $P(h,t)$, we transform the equation using the probability generating function $Q(s,t) = \sum_{h=0}^{\infty} P(h,t)s^h$. We have also neglected the subscript i because our model is "neutral" and $P(h,t)$ can describe the size of any HSC clone i. If the HSC self-renewal rate is approximated as $r_{\rm h}(H(t)) \equiv r_{\rm h}(t)$, the solution for $Q(s,t)$ takes on the following form [1]:

$$Q(s,t) = 1 - \frac{s-1}{(s-1)\phi(t) - \psi(t)}, \tag{A1}$$

where

$$\psi(t) = e^{-\int_0^t (r_{\rm h}(t') - \mu_{\rm h})\mathrm{d}t'} \ \text{ and } \ \phi(t) = \int_0^t r_{\rm h}(t')\psi(t')\mathrm{d}t'. \tag{A2}$$

Note that for $h \geq 1$,

$$Q^{(h)}(s,t) = \frac{\partial^h Q(s,t)}{\partial s^h} = \frac{h!(-\phi(t))^{h-1}\psi}{[(s-1)\phi(t) - \psi(t)]^{h+1}} \ \text{ and } \ P(h,t) = \frac{Q^{(h)}(0,t)}{h!} = \frac{\phi^{h-1}(t)\psi(t)}{(\phi(t)+\psi(t))^{h+1}}. \tag{A3}$$

These solutions obey the initial condition $P(h,0) = \mathbb{1}(h,1)$ and as $t \to \infty$, $\psi(t) \to \psi(\infty) \in (0,1)$, $\phi \to \infty$, and $P(h,t) \to 0$. For $h = 0$, $P(0,t) = 1 - \frac{1}{\phi(t)+\psi(t)}$ and $P(0, t \to \infty) \to 1$, indicating eventual extinction at long times [1, 2].

Using forms given in Eq. (A3), since both ϕ and ψ are independent of h, we can define

$$\frac{P(h+1,t)}{P(h,t)} = \frac{\phi(t)}{\phi(t)+\psi(t)} \equiv \lambda(t). \tag{A4}$$

Thus, the probability distribution $P(h,t)$ can be written as

$$P(h,t) = \frac{1}{\phi(t)+\psi(t)} \frac{\psi(t)}{\phi(t)+\psi(t)} \left(\frac{\phi(t)}{\phi(t)+\psi(t)}\right)^{h-1} = (1 - P(0,h))(1-\lambda(t))\lambda(t)^{h-1}. \tag{A5}$$

B Alternative model of progenitor aging

An alternative model to the one we have analyzed allows younger-generation progenitor cells ($\ell < L$) to differentiate into peripheral blood. Since each generation can differentiate with rate ω, the progenitor cell dynamics are slightly modified from those in our main model:

$$\frac{\mathrm{d}n^{(\ell)}(t)}{\mathrm{d}t} = \begin{cases} \text{Poisson}(\alpha h(t)) - (r_{\rm n} + \mu_{\rm n} + \omega)n^{(0)}(t), & \ell = 0, \\ 2r_{\rm n}n^{(\ell-1)}(t) - (r_{\rm n} + \mu_{\rm n} + \omega)n^{(\ell)}(t), & 1 \leq \ell \leq L-1, \\ 2r_{\rm n}n^{(L-1)}(t) - (\omega + \mu_{\rm n}^{(L)})n^{(L)}(t), & \ell = L. \end{cases} \tag{B1}$$

Moreover, the dynamics of the mature peripheral blood population obey

$$\frac{\mathrm{d}m(t)}{\mathrm{d}t} = \sum_{\ell=0}^{L} \omega n^{(\ell)}(t) - \mu_{\mathrm{m}} m(t). \tag{B2}$$

The solution to Eqs. (B1) and (B2) following a single differentiation event is

$$\begin{aligned} n_{\mathrm{b}}^{(\ell)}(t) &= \frac{(2r_{\mathrm{n}})^{\ell}}{\ell!} t^{\ell} e^{-(r_{\mathrm{n}}+\mu_{\mathrm{n}}+\omega)t}, \\ n_{\mathrm{b}}^{(L)}(t) &= e^{(r_{\mathrm{n}}-\mu_{\mathrm{n}}-\omega)t}\left[1 - \frac{\gamma(L+1, 2r_{\mathrm{n}}t)}{L!}\right], \\ m_{\mathrm{b}}(t) &= \omega \int_0^t \sum_{\ell=0}^{L} n_{\mathrm{b}}^{(\ell)}(\tau) e^{-\mu_{\mathrm{m}}(t-\tau)} \mathrm{d}\tau \end{aligned} \tag{B3}$$

These results can be applied to the model and analyzed and simulated using the same procedures as described in the main text. However, certain parameters have to be re-interpreted. For example, using the same value of $\omega = 0.16$ will significantly increase the effective death rate for progenitor cells of each generation. Fortunately, as we will show later, this alternative mechanism should not affect our main conclusion as the parameter-fitting results are not sensitive to the exact shape of cell bursts.

C Mean extinction time for a clone

As a function of the initial number h of HSCs in a clone, the mean extinction time (MET) $T(h)$ under the steady-state approximation $r_{\mathrm{h}} = \mu_{\mathrm{h}}$ obeys [3, 4]

$$[T(h+1) - T(h)]\mu_{\mathrm{h}} h - [T(h) - T(h-1)]\mu_{\mathrm{h}} h = -1. \tag{C1}$$

with an absorbing boundary condition $T(0) = 0$. By iterating Eq. (C1), we find

$$T(h+1) - T(h) = T(1) - \frac{1}{\mu_{\mathrm{h}}} \sum_{k=1}^{h} \frac{1}{k}, \tag{C2}$$

which can be again iterated to obtain

$$T(h) = hT(1) - \frac{1}{\mu_{\mathrm{h}}} \sum_{k=1}^{h-1} \sum_{\ell=1}^{k} \frac{1}{\ell}. \tag{C3}$$

To solve for $T(1)$, we invoke a reflecting boundary condition $T(H_{\mathrm{ss}}) - T(H_{\mathrm{ss}} - 1) = 1/(\mu_{\mathrm{h}} H_{\mathrm{ss}})$ [5], where

$$T(H_{\mathrm{ss}}) = H_{\mathrm{ss}} T(1) - \frac{1}{\mu_{\mathrm{h}}} \sum_{k=1}^{H_{\mathrm{ss}}-1} \sum_{\ell=1}^{k} \frac{1}{\ell}, \qquad T(H_{\mathrm{ss}} - 1) = (H_{\mathrm{ss}} - 1)T(1) - \frac{1}{\mu_{\mathrm{h}}} \sum_{k=1}^{H_{\mathrm{ss}}-2} \sum_{\ell=1}^{k} \frac{1}{\ell}, \tag{C4}$$

to find

$$T(1) = \frac{1}{\mu_{\mathrm{h}}} \sum_{\ell=1}^{H_{\mathrm{ss}}} \frac{1}{\ell}. \tag{C5}$$

Upon using Eq. (C5) in Eq. (C3), we find

$$T(h) = \frac{h}{\mu_{\mathrm{h}}} \sum_{k=1}^{H_{\mathrm{ss}}} \frac{1}{k} - \frac{1}{\mu_{\mathrm{h}}} \sum_{k=1}^{h-1} \sum_{\ell=1}^{k} \frac{1}{\ell} \equiv T_{\mathrm{discrete}}(h), \tag{C6}$$

which is the MET for a discrete system.

We can also approximate $T(h)$ by considering h as a continuous variable, and replace the summations in Eq. (C6) by integrations to find a simpler, more insightful approximation to $T(h)$:

$$\begin{aligned} T_{\text{continous}}(h) &= \frac{h}{\mu_{\text{h}}}\int_{\ell=1}^{H_{\text{ss}}}\frac{\mathrm{d}\ell}{\ell} - \frac{1}{\mu_{\text{h}}}\int_{k=1}^{h-1}\mathrm{d}k\int_{\ell=1}^{k}\frac{\mathrm{d}\ell}{\ell} \\ &= \frac{h\ln H_{\text{ss}} - (h-1)\ln(h-1) + h - 2}{\mu_{\text{h}}} \\ &\approx \frac{h}{\mu_{\text{h}}}\left(\ln\frac{H_{\text{ss}}}{h} + 1\right), \end{aligned} \tag{C7}$$

where we have used $\int^{x}(1/x')\mathrm{d}x' = \ln x$ and $\int^{x}\ln x'\mathrm{d}x' = x\ln x - x$. The continuous approximation to the MET matches the exact result quite well (relative error $\lesssim 5\%$) for all values of h.

D Effective parameters and symmetric HSC differentiation

There are differing reports on the measured death rates for circulating granulocytes. We have used the most recently reported value $\mu_{\text{m}} = 1$ per day for humans. The effect of changing the value of $\mu_{\text{m}} \to \mu'_{\text{m}}$ on our analysis is a reinterpretation of L_{e}. By rewriting Eq. (13) as $A^{+}_{\text{ss}}2^{L_{\text{e}}} = M^{+}_{\text{ss}}\mu_{\text{m}} = M^{+}_{\text{ss}}\mu'_{\text{m}}\left(\frac{\mu_{\text{m}}}{\mu'_{\text{m}}}\right)$, we rearrange the expression to $A^{+}_{\text{ss}}2^{L_{\text{e}}+\log_2(\mu'_{\text{m}}/\mu_{\text{m}})} = M^{+}_{\text{ss}}\mu'_{\text{m}}$ and find $L'_{\text{e}} = L_{\text{e}} + \log_2(\mu'_{\text{m}}/\mu_{\text{m}})$. For example, $\mu'_{\text{m}} = 2$ would lead to $L'_{\text{e}} = L_{\text{e}} + 1$, where one additional round of progenitor doubling compensates for the doubled loss rate of mature granulocytes. One may argue that the change in μ_{m} can also be compensated for by doubling A^{+}_{ss}, which would have a different effect on the burstiness of the model compared to doubling L_{e}. However, when re-fitting the data with $\mu'_{\text{m}} = 2$ or 0.2, we observed that $(A^{+}_{\text{ss}})^{*}$ did not change much, with most of the effect of modifying μ_{m} absorbed by changes in L^{*}_{e}.

Similarly, uncertainties in other parameters can also be subsumed into L_{e}. For example, setting $\mu_{\text{n}}^{(L)} = \omega > 0$ implies that only half of the generation-L progenitors contribute to the peripheral blood. For a model with $\mu_{\text{n}}^{(L)} = 0$ to generate an equivalent effect, we can halve the number of mature cells by using an effective maximum generation parameter $L'_{\text{e}} = L_{\text{e}} - 1$. This indicates that the intrinsic clone size fluctuations demonstrated in the experimental data strongly constrain A^{+}_{ss}.

Another possible modification of our mechanistic model is to allow for the possibility of symmetric HSC differentiation. The effect of symmetric differentiation can again be subsumed into the parameter L_{e} without qualitatively affecting our analysis. Assume a proportion $0 \le q \le 1$ of HSC differentiations are symmetric, producing on average $1+q$ generation-0 progenitor cells. After L_{e} rounds of proliferation, the $1+q$ generation-0 progenitors produce on average $(1+q)\times 2^{L_{\text{e}}}$ mature cells. This is equivalent to an exclusively asymmetric differentiation model ($q = 0$) with $L'_{\text{e}} = L_{\text{e}} + \log_2(q+1)$. We also expect symmetric differentiation to slightly increase the speed of coarsening since each HSC differentiation is also accompanied by the HSC's death and clones represented by a single HSC would disappear under symmetric differentiation. However, given the small rate α of HSC differentiation, the large number C_{h} of clones, and the insensitivity of our results to the distribution h_i, the data cannot quantitatively resolve the symmetric-asymmetric modes of HSC differentiation.

E Alternative objective functions and statistical insights

We developed our data analysis based on the statistics of the quantity y_i, the time averaged relative clone sizes for those clones exhibiting z absences across their longitudinal samples. While reasonable parameter estimates were obtained from fitting to data, we also considered alternative objective functions. Specifically, we looked at the standard deviation $\sigma_i = \sqrt{\frac{1}{J}\sum_{j=1}^{J}(f_i(t_j) - y_i)^2}$ quantifying the temporal fluctuations of the relative sizes of each clone i. The way we construct an alternative objective function is similar to the way we constructed Y_z. Recall for Y_z, we calculated the average abundance across only those clones with the same $z_i = z$ absences across time. However, unlike z_i which takes a finite set of discrete values $\{1,\ 2,\ ...,\ J-1\}$,

σ_i is a continuous variable so we have to artificially bin their values. Instead, we bin clones with similar y_i and study the average of their associated σ_i's. Since the distribution y_i is non-linear with a long tail, we evaluated $\ln y_i$ to obtain the near-linear distribution shown in Fig. E1(a), sorted $\ln y_i$ into equal-width bins, and calculated the average of the associated σ_is. Dividing the values of $\ln y_i$ into bins labeled by k, we compute

$$U_k = \frac{\sum_i \sigma_i \mathbb{1}(\text{clone}\, i \in \text{bin}\, k)}{\sum_i \mathbb{1}(\text{clone}\, i \in \text{bin}\, k)} \tag{E1}$$

in analogy with the definition of Y_z. The objective function can be straightforwardly defined as

$$\text{MSE}_\sigma(\theta_{\text{model}}) = \sum_k (U_k(\theta_{\text{model}}) - \hat{U}_k)^2. \tag{E2}$$

It is also unclear how to set upper and lower bounds on the range of y_i for comparison (in contrast to the natural bound on $1 \leq z \leq J-1$) because an unconstrained set of clones will be sensitive to the underlying h_i distribution (an undesirable property). In Fig. E1(b), we fit the data from animal RQ5427 using MSE_σ and find $L_{\text{e}}^* \approx 24.4$, consistent with our previous estimate using Y_z.

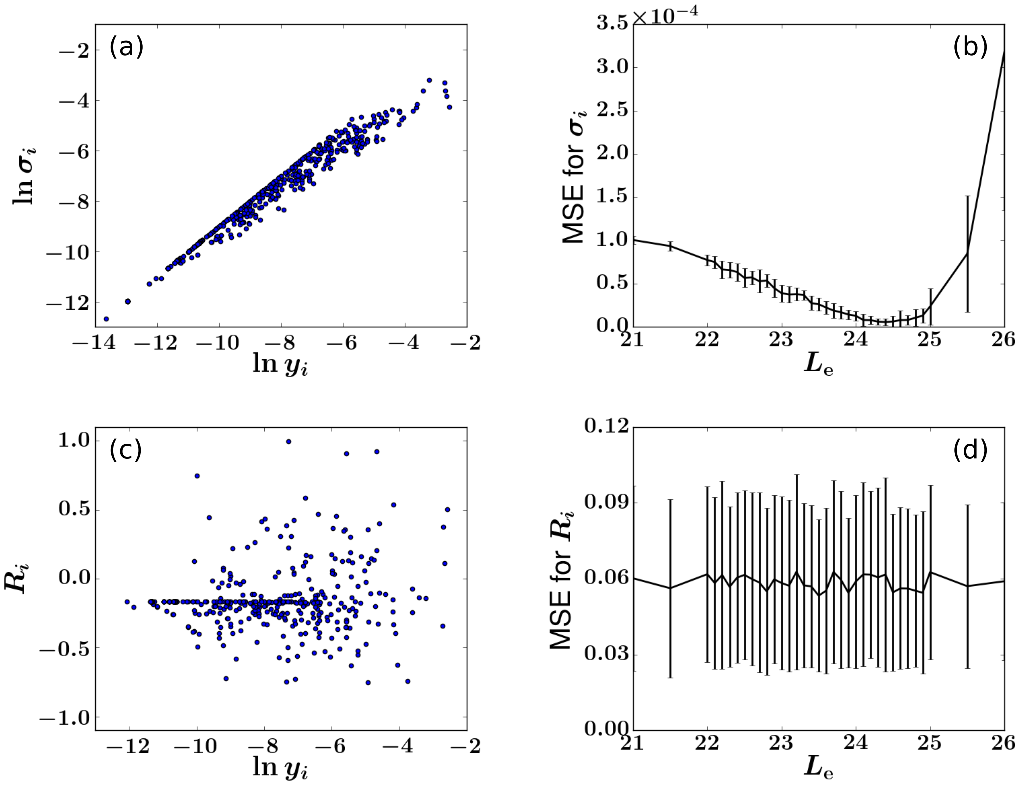

Figure E1: Statistics of the two alternative fluctuation measures and their fitting results. Each dot represents a clone. (a) Log standard deviation plotted against log average abundances. Clones are near-linearly distributed in the log average abundance space. (b) Objective function MSE_σ *vs.* L_{e}. Clones of similar y_i are binned, and their averaged σ_i were used to compute U_k. (c) Autocorrelations R_i *vs.* log of average abundances u_i. There is no clear pattern in the distribution of R_is. (d) MSE_R *vs.* L_{e}. This objective function cannot resolve the LSE L_{e}^*.

While it is also possible to choose σ_i as a measure of clone population fluctuations, we list several advantages of $\hat{z}_i$ over σ_i for the current dataset. Note that the number of disappearances z_i of each individual clone is defined on a finite set of integers (unlike the continuously measured σ_i), making it easier to bin clones

with the same z values. Different clones i will exhibit different time-averaged abundances y_i but may have the same value of z_i. As shown in Fig. 4 in the main text, the larger $\hat{z}_i$ is, the smaller the corresponding $\ln \hat{y}_i$ tends to be. The robust correlation between z_i and y_i encodes the level of fluctuations for a clone of certain size. For a given y_i, the larger z_i, the "burstier" the dynamics, implying a smaller number of tagged HSC differentiations per unit time (a smaller $A_{\rm ss}^+$).

Another advantage of using z_i statistics emerges when fitting model results to the pattern of the measured data in Fig. 4 in the main text. Average sizes y_i (and the underlying h_i) associated with clones having $1 \leq z \leq 7$ all contain at least one absence. This constraint naturally controls the upper and lower bounds of h_i in a particular z bin ($1 \leq z \leq 7$), based on the burstiness of the model. Exact knowledge of the configuration $\{h_i\}$ is not required for fitting these y_i data.

Thus, dividing clones into z bins provides us with a natural way to exclude unconstrained clone sizes. In other words, the theoretical values of y_i (and the underlying h_i) associated with bin $z_i = 0$ can be arbitrarily and unreasonably large, and such a possibility should be excluded. Similarly, all y_i below a threshold size generate $z_i = J$ (clones that never appeared in the sampled blood) and do not provide any statistical power. This advantage of using z_i can also be confirmed by visual inspection of Fig. 9(b) in the main text. Several very large clones do not follow the general statistical pattern and show extremely large variances. Without manually filtering out these clones, our fitting in Fig. 1(b) results in a larger $L_{\rm e}^* = 24.4$ than the $L_{\rm e}^* = 23.4$ obtained in the main text using Y_z statistics. Finally, another option for comparing model with data is to use correlation functions. In this approach, the sampling gap Δt_j varies between 5 and 11 months, so the usual autocorrelation function with equal time gaps cannot be rigorously defined. We use the one-sample-gap autocorrelation function

$$R_i = \frac{1}{(J-1)\sigma_i^2} \sum_{j=1}^{J-1} (f_i(t_j) - y_i)(f_i(t_{j+1}) - y_i) \tag{E3}$$

and bin values of $\ln y_i$ in analogy to Eq. (E1) to define

$$W_k = \frac{\sum_i R_i \mathbb{1}(\text{clone}\, i \in \text{bin}\, k)}{\sum_i \mathbb{1}(\text{clone}\, i \in \text{bin}\, k)} \tag{E4}$$

and construct an autocorrelation-based objective function

$$\text{MSE}_R(\theta_{\rm model}) = \sum_k (W_k(\theta_{\rm model}) - \hat{W}_k)^2. \tag{E5}$$

Since the inter-sample intervals Δt_j are larger than a typical burst size $\Delta\tau_{\rm b} \approx 32$ days, cells in different samples likely originate from different HSC differentiation events. Thus, the fluctuations of clone sizes are uncorrelated from sample to sample, as shown in Fig. E1(c). Randomly distributed between -1 and 1, the values of R_i are centered about the line $R = \frac{1}{2-J}$, corresponding to the majority of clones that have $z_i = J-1$ (only 1 non-zero sample). Data fitting using R_i and MSE_R is ill-conditioned and cannot resolve $L_{\rm e}^*$, as shown in Fig. E1(d).

F Simulation of the forward model

To generate predictions, we first choose values of $\theta_{\rm model} = \{\lambda, C_{\rm h}, r_{\rm n}, L_{\rm e}\}$ and simulate our model, including sampling, to find $s_i(t_j)$. To simulate each realization of our model we

1. Specify the static HSC clone size distribution $P(h)$ by choosing the pair $(\lambda, C_{\rm h})$ and draw $\{h_i\}$ from the geometric distribution $C_{\rm h}$ times using the Python package np.random.geometric. Normalize to construct the configuration $\{h_i\}/H_{\rm ss}^+ \equiv \{\frac{h_i}{\sum_{i=1}^{C_{\rm h}} h_i}\}$. Alternatively, we can also use the data $\hat{y}_i$ to approximate the configuration $\{h_i\}/H_{\rm ss}^+$.

2. Fix all parameters $\theta_{\rm model}$, construct the total clone i differentiation rate $\alpha h_i \equiv A_{\rm ss}^+ \frac{h_i}{H_{\rm ss}^+} = 2^{-L_{\rm e}} \hat{M}_{\rm ss}^+ \mu_{\rm m} \frac{h_i}{H_{\rm ss}^+}$ for each clone i. Generate realizations of sets of HSC differentiation event times $\{\tau_k^{(i)}\}$ for each clone i based on the rate $\alpha h_i = A_{\rm ss}^+ h_i / H_{\rm ss}^+$.

3. Evaluate Eqs. (10) and (11) in the main text. Sum up the peripheral blood bursts initiated by each differentiation event of each clone i to find $m_i(t) = \sum_k m_{\rm b}(t - \tau_k^{(i)})$.

4. Sample a fraction $\varepsilon(t_j) = \frac{\hat{S}^+(t_j)}{\hat{M}^+(t_j)}$ of the total peripheral cell count $M^+(t_j) = \sum_i m_i(t_j)$. Here, $\hat{S}^+(t_j)$, $\hat{M}^+(t_j)$, and the times t_j are defined by the experiment. We used the Python package numpy.random.binomial. The cell counts of each clone are $s_i(t_j)$. Use the simulated total tagged cell counts in the samples $S^+(t_j) = \sum_i s_i(t_j)$ to normalize $\frac{s_i(t_j)}{S^+(t_j)} = f_i(t_j)$. Up to this point, we have generated a data matrix $f_i(t_j)$ of size $C_{\rm h} \times J$.

5. Increment $L_{\rm e}$ within the desired interval and repeat steps 2-4 200 times. For each value of $L_{\rm e}$, the 200 simulations generate 200 $f_i(t_j)$ matrices. These repeats are to ensure that the noise induced from drawing values of h_i from $P(h)$ and sampling $s_i(t_j)$ from $m_i(t_j)$ do not significantly corrupt our parameter estimation.

The simulated, model-derived configurations $f_i(t_j)$ are then compared with experimentally measured values $\hat{f}_i(t_j)$. The parameter $L_{\rm e}$ that minimizes the mean-squared error will be chosen as the least-squares estimate $L_{\rm e}^*$.

G Robustness to samping frequency and threshold

The robustness of our inference of $L_{\rm e}^*$ to sampling frequency is demonstrated for animal RQ5427 by excluding some time samples. In Figs. G1(a-h), we plot the MSE function by including only the first $j = (8, 7, \ldots, 1)$ time samples of the data. In this data set (animal RQ5427), the MSE remains meaningful, and the reconstruction of $L_{\rm e}^*$ is unchanged as long as at least four or five time samples are used. This conclusion is independent of which sampling time points are excluded. Since the system is well-approximated by a statistical steady state, the key determinant for robust inference is the *number* of samples included in the analysis.

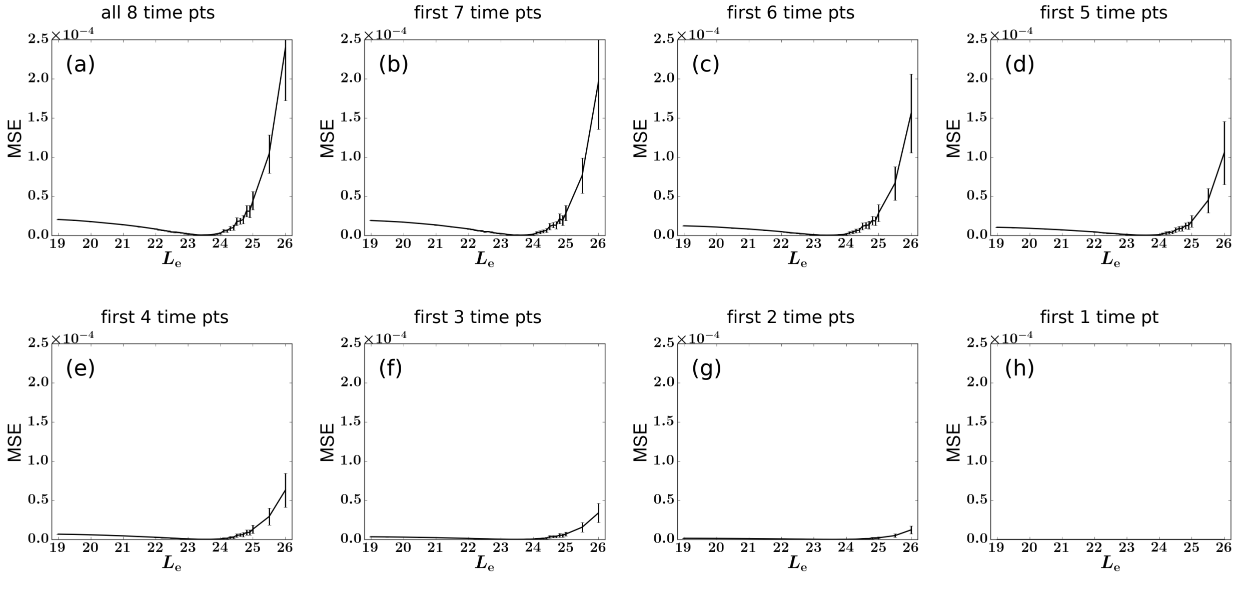

Figure G1: Simulated MSEs with $\lambda = 0.99, C_{\rm h} = 500, r_{\rm m} = 2.5$ for different numbers of time samples. From (a-h), only the first $j = (8, 7, \ldots, 1)$ time samples are used to fit the model. Provided at least two time samples are used, the reconstruction of $L_{\rm e}^* \approx 23.4 - 23.6$ remains fairly robust.

Robustness to a larger threshold of clone sizes is also demonstrated by eliminating clones whose average abundances are under a certain threshold in both the experimental and simulated data. In Figs. G2(a-h),

we plot the MSE corresponding to the clone frequency thresholds $1.16 \times 10^{-5}, 2.03^{-5}, 3.41 \times 10^{-5}, 8.84 \times 10^{-5}, 1.66 \times 10^{-4}, 3.30 \times 10^{-4}, 6.78 \times 10^{-4}, 1.46 \times 10^{-3}$, respectively. Using these thresholds, the numbers of clones retained in the analysis are 482, 428, 375, 322, 268, 215, 159, and 107, corresponding to 90%, 80%, 70%, 60%, 50%, 40%, 30%, and 20% of the 536 total number of clones detected in animal RQ5427. Figs. G2 show that as long as $\gtrsim 200$ clones are included (a-f), the MSE yields a clear LSE $L_{\rm e}^* = 23.4 - 23.6$. Only at very high thresholds, where only 20-30% of the clones are retained, does the minimum of the MSE shift to slightly higher values $L_{\rm e}^* \approx 23.8, 24.3$ as shown in Figs. G2(g-h), respectively. Thus, we conclude that the inference of $L_{\rm e}^*$ from the data is fairly insensitive to sampling threshold provided a reasonable number of clones (typically $\gtrsim 200$) are included in the analysis.

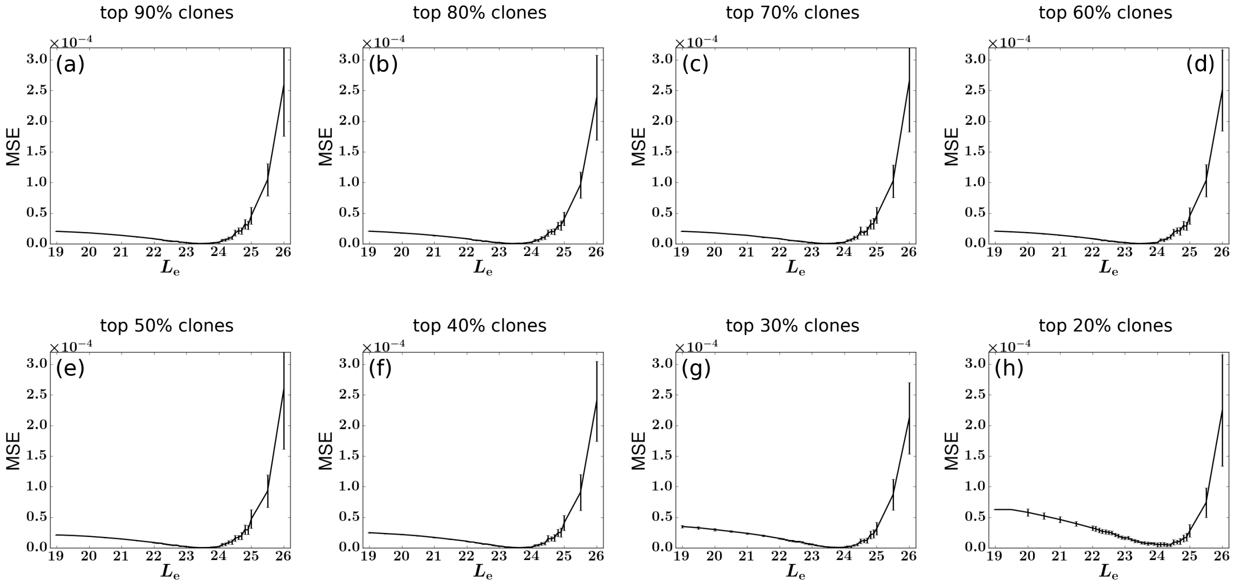

Figure G2: MSEs for animal RQ5427 using successively higher clone detection thresholds. Unique reconstruction of $L_{\rm e}^*$ is robust (a-f) even if only 40-50% of the clones are counted. (g-h) At even higher thresholds, the LSE for $L_{\rm e}^*$ increases only very slightly.

References

[1] Wang M. Nonhomogeneous Birth-death Processes, M. S. Thesis: California State Polytechnic University, Pomona. M. S. Thesis: California State Polytechnic University, Pomona; 2005.

[2] Yang J, Sun Z, Komarova N. Analysis of stochastic stem cell models with control. Mathematical Biosciences. 2015;266:93–107.

[3] Gardiner CW. Handbook of Stochastic Methods: For physics, chemistry, and natural sciences. Springer, Berlin; 1985.

[4] Allen L. An Introduction to Stochastic Processes with Applications to Biology. Taylor and Francis; 2010.

[5] Doering CR, Sargsyan KV, Sander LM. Extinction Times for Birth-Death Processes: Exact Results, Continuum Asymptotics, and the Failure of the Fokker–Planck Approximation. Multiscale Modeling & Simulation. 2005;3(2):283–299.
